# The Accidental Factor in Mortality

**Published:** 1915-09

**Authors:** 


### The Accidental Factor in Mortality.

The recent overturning of an excursion steamer, almost at the dock, in Chicago, with a loss of life of approximately 1500, is said to make a new record in drowning mortality, exceeding that of the Titanic disaster or that due to the sinking of any other boat, at any time, from any cause whatever, even including naval warfare. The wreck of a troop train in England, while in a sense, a military casualty, was essentially due to the same errors usually considered to affect this country and not those of Europe. The destruction of about 40 lives in Erie, by drowning and traumatisms due to a cloud burst overflowing a small creek was one of the least foreseeable disasters on a large scale reported since the California earth-quake. Meantime, N. Y. City reports an increase of automobile casualties and an expected death list for the year of about 400.

Under present conditions, in this country, suicide accounts for about 1.1% of all deaths—perhaps more, from the tendency to ascribe to accidents, deaths really suicidal—while other violent and accidental deaths not due to disease, account for 6.4% of all deaths. Thus it will be seen that at least 7.5% of all deaths are theoretically preventable, without regard to hygiene, sanitation, or skill in treating disease. As improvement in these factors progresses, this percentage is likely to be increased. In almost all discussions of the death rate, the medical profession is, implicitly or explicitly, held responsible for the total though, obviously, in no personal sense except by extremists.

We make no reference at present to military mortality. Enormous as this is, it is by no means so important a factor as generally considered. During the Civil War, it was about 20:1000 per year, less than the average aggregate peace mortality of the period though, of course, higher than the death rate of healthy adult males—the class from which soldiers are mainly drawn. Statistics for the present war are so incomplete that it is impossible to make any definite statement though the opinion may be hazarded that the increased destructiveness of modern weapons is more than balanced by the general use of trenches, not to speak of improved sanitation.

